# Trans‐spinal direct current stimulation modifies spinal cord excitability through synaptic and axonal mechanisms

**DOI:** 10.14814/phy2.12157

**Published:** 2014-09-28

**Authors:** Zaghloul Ahmed

**Affiliations:** 1 Department of Physical Therapy, College of Staten Island for Developmental Neuroscience, the College of Staten Island, Staten IslandNew York, New York; 2 Graduate Center/The City University of New York, New York, New York

**Keywords:** Direct current, excitability, trans‐spinal

## Abstract

The spinal cord is extremely complex. Therefore, trans‐spinal direct current stimulation (tsDCS) is expected to produce a multitude of neurophysiological changes. Here, we asked how tsDCS differentially affects synaptic and nonsynaptic transmission. We investigated the effects of tsDCS on synaptically mediated responses by stimulating the medullary longitudinal fascicle and recording responses in the sciatic nerve and triceps and tibialis anterior muscles. Response amplitude was increased during cathodal‐tsDCS (c‐tsDCS), but reduced during anodal‐tsDCS (a‐tsDCS). After‐effects were dependent on the frequency of the test stimulation. c‐tsDCS‐reduced responses evoked by low‐frequency (0.5 Hz) test stimulation and increased responses evoked by high‐frequency (400 Hz) test stimulation. a‐tsDCS had opposite effects. During and after c‐tsDCS, excitability of the lateral funiculus tract (LFT) and dorsal root fibers was increased. However, a‐tsDCS caused a complex response, reducing the excitability of LFT and increasing dorsal root fiber responses. Local DC application on the sciatic nerve showed that the effects of DC on axonal excitability were dependent on polarity, duration of stimulation, temporal profile (during vs. after stimulation), orientation of the current direction relative to the axon and relative to the direction of action potential propagation, distance from the DC electrode, and the local environment of the nervous tissue. Collectively, these results indicate that synaptic as well as axonal mechanisms might play a role in tsDCS‐induced effects. Therefore, this study identified many factors that should be considered in interpreting results of DCS and in designing tsDCS‐based interventions.

## Abstract (precis)

There are two plastic mechanisms operating in different regions in the nervous system: synaptic‐mediated mechanisms and intrinsic excitability mechanisms. This study indicates that direct current stimulation affects both synaptic and intrinsic mechanisms of plasticity.

## Introduction

Trans‐spinal direct current stimulation (tsDCS) modulates the activity of spinal pathways and circuits in both humans and animals. The main goal of tsDCS is to ameliorate disease sequelae locally at the spinal cord (e.g., spinal cord injury) or brain (e.g., stroke). Studies have shown that tsDCS can exert polarity‐dependent quantitative control over somatosensory inputs in rats (Aguilar et al. 2011) and humans (Cogiamanian et al. 2008), motor cortex output in mice (Ahmed 2011; Ahmed and Wieraszko 2012) and humans (Lim and Shin 2011), and spinal reflexes in healthy humans (Winkler et al. 2010; Lamy et al. 2012) and those with spinal cord injury (Hubli et al. 2013). In addition, our laboratory showed that tsDCS could induce qualitative (i.e., rhythmicity) and quantitative (i.e., amplitude) changes in spinal circuit activity (Ahmed 2013a) and qualitative improvements in skilled locomotion (Ahmed 2013b). Recent studies showed that peripheral nerves are affected by tsDCS \(Ahmed 2014; Parazzini et al. 2014) and transcranial DC stimulation (Ardolino et al. 2005; Di Lazzaro et al. 2013). Thus, comprehensive evaluation of the effects of DCS on axonal excitability is warranted.

Clinical optimization of tsDCS requires a better understanding of its short‐ and long‐term effects on neural function, as well as its behavioral consequences. In this study, we asked basic albeit critical questions: how does tsDCS affect the activation threshold of white matter (axons)? How does it modulate synaptic‐mediated responses? Are its effects polarity‐dependent? The answers to these questions will form the experimental foundation to guide and broaden tsDCS research and clinical applications. In addition, determining rules of stimulation by quantifying the effect of DCS on neural systems will allow scientists and clinicians to predict behavioral effects based on DC location, magnitude, orientation, and polarity. An important step toward establishing rules of tsDCS is to test its specific effects on synaptic transmission compared to effects on axonal excitability. Thus, in this study, we hypothesized that tsDCS would differentially affect synaptically and nonsynaptically mediated spinal responses.

To assess synaptically mediated activity, we recorded from the sciatic nerve and associated muscles in response to electrical stimulation of the medullary longitudinal fascicle (MLF). We also studied the effects of tsDCS on activation of the spinal lateral funiculus tract (LFT). Finally, a simpler sciatic nerve preparation was used to further characterize the effect of subthreshold DCS on axonal excitability. Together, these investigations revealed complex responses of spinal and peripheral neural tissue to DCS.

## Materials and Methods

### Animals

This study used adult male CD‐1 mice (*n* = 103; 35–40 g), which were housed under a 12:12‐h light–dark cycle with free access to food and water. Experiments were carried out in accordance with the National Institutes of Health Guidelines for the Care and Use of Laboratory Animals. Protocols were approved by the Institutional Animal Care and Use Committee of the College of Staten Island.

### Surgical procedures

The surgical procedure was performed as described previously (Ahmed 2011). Briefly, animals were anesthetized using ketamine/xylazine (90/10 mg/kg, i.p.). To maintain a moderate to deep level of anesthesia, muscle, and nerve activity were monitored throughout all experiments.

Animals were placed in a mouse stereotaxic apparatus. The bones at the base of the tail, distal end of the femur, and paw were fixed to the system's base with surgical pins. Incisions were made in the skin covering the hind limb, and the skin was moved to the side and held with clips. To monitor muscle activity during anesthesia in the sciatic nerve experiments, muscle isometric tension was recorded from the triceps muscles (TS). TS were carefully separated from the surrounding tissue. The TS tendon was threaded with a hook‐shaped 0–3 surgical silk, which was then connected to force transducers. Muscle length was adjusted to yield maximal response. In studies testing the LFT and MLF, muscle force was recorded from two muscles: tibialis anterior (TA) and triceps surae (TS). Tissue surrounding the distal part of the sciatic nerve was removed.

### Experiment 1: MLF testing

**Figure 1.**
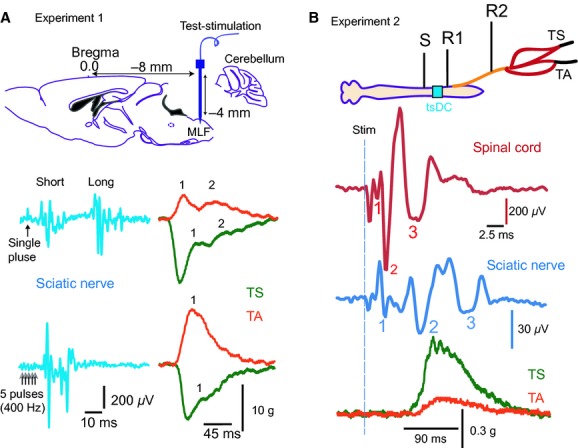
Experimental setup (MLF and LFT). (A) Experiment 1 setup. Top panel: Schematic illustration of MLF stimulation (8‐mm posterior and 4‐mm ventral from bregma). Middle panel: Examples of sciatic nerve responses evoked by a single 0.5 mA pulse applied at the left MLF. Note that two responses were distinguished: short and long latency. Bottom panel: Examples of sciatic nerve potentials evoked by stimulating the left MLF with a train of five pulses (0.2 mA; 400 Hz). Right: Examples of muscle twitches simultaneously recorded from left TA and TS muscles. Two twitches (1 and 2) were evoked in response to a single‐pulse test stimulation despite inhibition of TS muscle tension. High‐frequency test stimulation of LMF produced a single twitch in TA muscle (1) and reduced TS muscle tension, on which a twitch (1) was superimposed. (B) Experiment 2 setup: Stimulation (S) was delivered at the LFT, and recording sites were at LFT (R1) and the sciatic nerve (R2). The tsDC electrode location is colored cyan. Muscle twitch force was recorded from TS and TA. Bottom: Examples of spinal tract potential trace (red) and sciatic nerve potential trace (blue). Waves are numbered. Lower traces represent the concurrent twitch forces recorded form TS and TA muscles.

Animals were placed in the stereotaxic frame, and a large craniotomy was made to expose the cerebellum. The cerebellum was removed to allow access to the brain stem (Fig. 1A). Relative to bregma, MLF was located 8.0 mm posterior and 4.0 mm ventral from the skull surface. In these experiments, muscle force was recorded from TA and TS muscles. Sciatic nerve potentials were simultaneously recorded. Two stimulation paradigms were used to test the effects of tsDCS on MLF‐evoked spinal responses. In one paradigm, low‐frequency stimulation with a train of five pulses (intensity, 0.5 mA; duration, 0.1 ms; frequency, 0.5 Hz) was used to evoke spinal responses. In the second paradigm, high‐frequency stimulation with a train of five pulses (intensity, 0.2 mA; duration, 0.05 ms; frequency, 400 Hz) was used. Short‐ and long‐term effects of tsDCS on MLF‐evoked spinal responses were tested in separate cohorts of animals. Biphasic test stimulation was delivered before tsDCS, immediately after tsDCS onset, and immediately, 10 min, and 20 min after tsDCS offset using the PowerLab system and a stimulus isolator unit (FE180), which produces constant‐current pulses. The DC electrode was placed on the vertebral column between T13 and L5. tsDCS was delivered using a Grass stimulator and constant current isolation unit (Grass Technologies, West Warwick, RI). In all experiments, tsDCS was ramped for 10 sec, and amplitude was 0.8 mA.

### Experiment 2: LFT testing

Two distinct laminectomies were performed to expose the spinal cord at T9 and L6. A spinal DC electrode (width, 4 mm; length, 6 mm) was placed over the intact spinal column region between those two laminectomies (Fig. 1B). tsDCS was delivered using a Grass stimulator and constant current isolation unit (Grass Technologies). In all experiments, tsDCS was ramped for 10 sec, and amplitude was 0.8 mA. Care was taken to widen the size of the laminectomies to expose the LFT, which was clearly identified as a distinct, white‐colored band located lateral to the grayish dorsal horn. Test stimulation (five pulses; frequency, 0.5 Hz; intensity, 0.2 mA; duration, 0.2 ms) was delivered above dura by a concentric electrode (outer pole, 250 *μ*m (31 ga); tip, rounded (standard); inner pole, 125 *μ*m), which was selected instead of a monopolar electrode to reduce interference with the tsDCS electrode. The stimulator consisted of a PowerLab system and stimulus isolator unit (FE180), which produces constant‐current pulses. Recording electrodes were placed on the LFT at L6 and on the sciatic nerve. Muscle twitch force was simultaneously recorded from TS and TA muscles. Test stimulation was performed before, during, immediately after, and 10, 20, 30, and 40 min after tsDCS offset.

Differential recordings for MLF and LFT were collected using a NeuroAmp Ex Headstage. The headstage has three input connections: single input (+), single input (−) or reference, and ground. The reference electrode was inserted in the adjacent abdominal skin on the left side of the body. The ground electrode was attached to the contralateral abdominal skin (right side of the body). The signal was filtered (bandpass, 100 Hz‐ 2 kHz), digitized at 4 kHz, and stored in the computer for further processing. A power lab data‐acquisition system and LabChart 7 software (AD Instruments) were used to acquire and analyze the data.

### Experiment 3: Sciatic nerve testing

#### Single‐electrode procedure and experimental design

**Figure 2.**
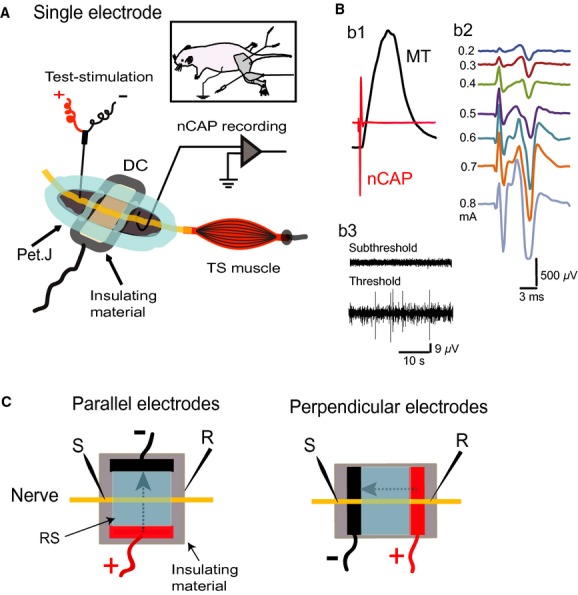
Experiment 3 setup (sciatic nerve). A. Single electrode: the DC electrode (DC) was a flat stainless steel plate (width, 7 mm) that was glued onto insulating material (silicone rubber). A reference electrode (Ref.) was attached to a flap of abdominal skin (insert). A hook electrode, placed between the muscle (3 mm) and the DC electrode (4 mm), was used to record nerve CAP (nCAP). The reference electrode was a needle inserted into the hindpaw skin. A bipolar concentric stimulating electrode was located 4 mm from the DC electrode. The nerve underneath both the stimulating and recording electrodes was placed on insulating material. Petroleum jelly (Pet.J) was mixed with silicone oil and applied, as seen in A, to create three isolated chambers: stimulating electrode chamber, DC electrode chamber, and recording electrode chamber. B. b1 is an example of a recording that includes an overlay of muscle twitch (MT) and nerve CAP (nCAP). b2 shows a graded nCAP series with increasing intensity to test stimulation current; therefore, the CAP amplitude was used as an indicator of nerve excitability. b3 is an example of nerve activity during DC application. DC was kept below threshold. C. Parallel electrodes: The general setup is similar to the single‐electrode setup, except that DC was applied using two plates running parallel to the nerve. The plates (length, 7 mm) were glued to silicone rubber, and petroleum jelly was used to create a chamber connecting the two plates. This chamber was filled with Ringer solution (RS). Great care was taken to keep DC electrodes completely insulated from the animal's body. Perpendicular electrodes: DC plates (width, 3.5 mm) were placed perpendicular to the nerve.

To test the effect of subthreshold direct current stimulation (subDCS) on local excitability of the sciatic nerve, we first used a single DC electrode configuration. Specifically, one electrode was placed underneath the sciatic nerve, and the reference electrode was connected to a flap of abdominal skin, as shown in Figure 2A. The single DC electrode was a stainless steel plate (thickness, 5 *μ*m; width, 7 mm; length, 15 mm). In exploratory experiments, we determined that the width of the DC electrode, not its length, was crucial to produce consistent results. Electrodes narrower than 7 mm were not effective to produce the effect observed in this study. The electrode was glued on top of a piece of silicone rubber (Fig. 2A), which was shaped to fit the area of the exposed sciatic nerve and stabilized by fixing it to the base using surgical pins. The silicone rubber served to insulate the electrode and the exposed area of the sciatic nerve from the rest of the body. The sciatic nerve was laid straight on the plate; it is important to create no bends in the nerve since this can change the current–nerve relationship. Petroleum jelly, combined with silicone oil to create tighter seals, was applied on all exposed tissue to create a chamber around the centre of the DC electrode. In these experiments, the chambers were filled with Ringer's solution. The DC reference electrode was an alligator clip that was attached to the abdominal skin. A concentric bipolar stimulating electrode (tip, 250 *μ*m) was used to stimulate the sciatic nerve. This electrode was located 4‐mm rostral to the DC electrode. The recording electrode was a hook electrode that was custom‐made of tungsten (resistance, 2 MΩ) and attached to the nerve about 3‐mm distal to the DC electrode. The reference electrode was a needle electrode inserted into a flap of the skin in the left hindlimb paw. The ground electrode was attached to the abdominal skin on the right side of the body. The signal was passed to a differential amplifier with an active headstage (DP‐311, Warner Instruments), filtered (bandpass, 100 Hz‐5 kHz), digitized at 4 kHz, and stored in the computer for further processing. A power lab data‐acquisition system and LabChart 7 software (AD Instruments) were used to acquire and analyze the data. Sciatic nerve stimulation was performed using a Digitimer DS7AH constant current stimulator (Digitimer Ltd., UK). A Grass stimulator and a constant current isolation unit (Grass Technologies, West Warwick, RI) were used to deliver subDCS. In all experiments, subDCS was ramped for 10 sec.

Given the width of the plate electrode and the 1‐mm diameter of the proximal sciatic nerve, current density produced by 10‐*μ*A current was 0.0014 A/M^2^ or 1.4 mA/cm^2^. Threshold was determined by gradually increasing DC strength until nerve spike activity began to appear, as shown in Figure 2B. Current intensity used in this study was below this threshold. Two test stimulation sites were used for subDCS studies: one site was at the nerve part facing the DC electrode and the other was proximal to the DC electrode. The maximal compound action potential (CAP) was determined at the beginning of each experiment and was defined as the strongest nerve response before the appearance of multiple spikes (Fig. 1B). Exploratory experiments revealed that cathodal stimulation reduced CAP and anodal stimulation increased it. Thus, to examine the extent of these effects, the baseline CAP was adjusted to about 25–30% of maximal before a‐subDCS testing and to about 80% of maximal before c‐subDCS testing. In long‐term experiments, separate cohorts of animals were used to assess the effects of test stimulation at: the nerve segment proximal to the anodal electrode (*n* = 7) or cathodal electrode (*n* = 6) and the nerve segment facing the DC anodal electrode (*n* = 6) or cathodal electrode (*n* = 5). Baseline CAP amplitudes were identical in groups for which the same polarity was tested.

To test reversibility of subDCS effects, 10 animals (*n* = 5/group) were used. In one group, cathodal sub‐DC (−10 *μ*A) was applied for 3 min to induce long‐lasting inhibition of nerve excitability, followed 10 min later by anodal sub‐DC (+10 *μ*A) applied for 3 min. In a second group, a‐subDCS was applied first, followed 10 min later by cathodal sub‐DC. In these experiments, only proximal test stimulation was used.

#### Parallel subDCS procedure and design

Two stainless steel plates (width, 4 mm; length, 7 mm; thickness, 250 *μ*m) were used (Fig. 2C). It is important to note that the length and thickness of the plates determine the area of sciatic nerve exposed to the electrical current. The distance between these two electrodes was 6 mm. Except for the edge facing the nerve, all other sides of the plate were painted with liquid tape to insulate it from the rest of the chamber. The two plates were glued into a silicone rubber sheet. Petroleum jelly and silicone oil mixture was used to create a chamber around the area between the plates. To isolate the effects of subDCS on the nerve trunk, great care was taken to ensure that DC electrodes were completely insulated from the body, the site of recording, and the site of test stimulation. Any unintended connection was sufficient to alter the effects of subDCS. For example, when a Ringer solution leak occurs between the central chamber and the body, subDCS effects of proximal test stimulation have a similar profile to those observed during between‐electrode test stimulation.

Four groups of animals were used to test the short‐term effects of subDCS in a parallel electrode arrangement. Currents were passed in either the lateral to medial or medial to lateral direction. Intensities, tested in a pseudorandom order for each direction, were 10, 15, and 20 *μ*A. Proximal test stimulation was used when the nerve was centered (about 2.5 mm from each electrode, *n* = 6) and when the nerve was brought closer to one of the electrodes (1 mm from closer electrode, 4 mm from further electrode, *n* = 5). Similarly, between‐electrode test stimulation was used when the nerve was centered (*n* = 5) and when the nerve was brought closer to one of the electrodes (*n* = 5). Long‐term effects of this arrangement were tested in three groups of animals: (1) proximal test stimulation was used when current was passed lateral to medial with the nerve centered between the two electrodes (n = 6), and (2–3) between‐electrode test stimulation was used when the nerve was brought closer to the cathode (*n* = 5) or anode (*n* = 5).

#### Perpendicular subDCS procedure and design

Two stainless steel plates (width, 3.5 mm; length, 15 mm; thickness, 50 *μ*m) were aligned normally relative to the sciatic nerve and glued to a piece of silicone rubber 5 mm apart (Fig. 2C). The sciatic nerve was placed straight and perpendicular to the two plates. Once the stimulating and recording electrodes were attached to the nerve, a mixture of petroleum jelly and silicone oil was used to cover the exposed tissue.

Four groups of animals were used to test the long‐term effects of perpendicular electrode subDCS arrangement. Proximal test stimulation was used to test distal to proximal subDCS effects (*n* = 6) and proximal to distal subDCS effects (*n* = 5). Similarly, between‐electrode test stimulation was used to test distal to proximal subDCS effects (*n* = 5) and proximal to distal subDCS effects (*n* = 5).

### Statistical analyses

Changes in sciatic nerve responses were evaluated using repeated measures (RM) ANOVA with a Holm–Sidak post hoc correction to test differences across time points. Independent variables were time course and stimulation condition; dependent variables were muscle force, nerve CAP amplitude, and nerve response latency. Pearson correlations were used to test correlations between sciatic nerve or spinal potentials (waves) and muscle twitch force. Statistical analyses were performed using SigmaPlot (SPSS). Muscle force, sciatic nerve potentials, and spinal potentials were measured using LabChart software (ADInstruments). Data in all graphs represent means ± SEM, except latency data, which represent medians. The critical level of significance was set at *P* < 0.05.

## Results

### Experiment 1: tsDCS modified evoked spinal output by electrically stimulating MLF

**Figure 3.**
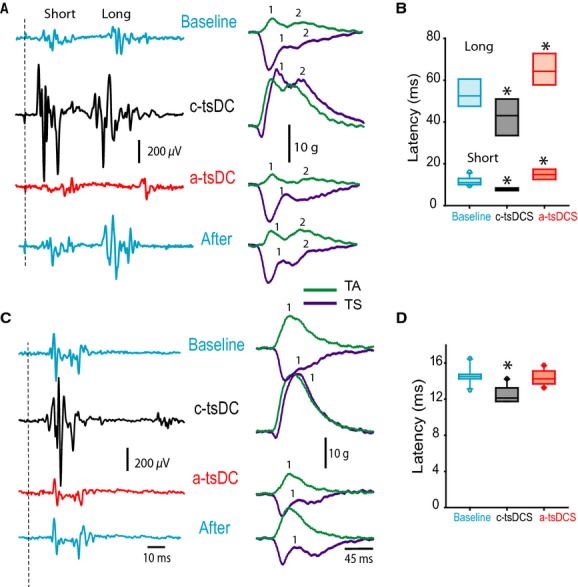
Immediate effects of tsDCS on MLF sciatic nerve‐evoked responses. (A) Single pulse. Left panel: Examples of sciatic nerve responses recorded before stimulation (blue, baseline), during c‐tsDCS (black) and a‐tsDCS (red), and immediately after offset (blue). Right: Concurrent muscle responses. Note that c‐tsDCS increased while a‐tsDCS decreased sciatic nerve potentials and concurrent muscle twitches. Numbers mark the two muscle twitches (1 and 2). Note that TS twitch 2 was not evoked during a‐tsDCS. (B) Box plot showing latency changes of short‐ and long‐latency sciatic nerve potentials. Short‐ and long‐latency responses were shortened during c‐tsDCS and prolonged during a‐tsDCS. (C) High frequency train (five pulses). Left: Sciatic nerve potential was significantly increased during c‐tsDCS and significantly decreased during a‐tsDCS. Right: Concurrent muscle twitches. (D) Box plot showing latency of sciatic nerve potentials. c‐tsDCS significantly shortened latency, but a‐tsDCS had no effect. *P < 0.01.

Mechanisms of synaptic transmission interact with the pattern of activity. High‐frequency stimulation induces long‐term potentiation, and low‐frequency stimulation induces long‐term depression of synaptic responses (Pockett and Figurov 1993; Shypshyna and Veselovs'kyi 2013). Experiment 1 aimed to test the effects on synaptic spinal response of: (1) tsDCS, and (2) the interaction between tsDCS and the pattern of test stimulation. Two paradigms of stimulation were used. Low frequency‐stimuli (five pulses, 0.5 Hz) applied at MLF elicited two distinguishable sciatic nerve potentials (with short‐ and long‐latency) and two corresponding muscle twitches (Fig. 1A). Consistent with previous findings (Ahmed 2011), during short tsDCS (10 sec), low‐frequency a‐tsDCS caused depression, whereas c‐tsDCS caused amplification of MLF‐evoked sciatic nerve responses (Fig. 3A). Sciatic nerve potentials and concurrent TA and TS muscle twitches returned to baseline levels after tsDCS offset. Note that during baseline, TS tension was reduced for about 30 ms before a small twitch appeared. During c‐tsDCS, the inhibition period was shortened to only about 8 ms before the TS was fully active. During a‐tsDCS, TS tension was reduced, but the twitch was slightly higher than baseline, and no second twitch was visible. This was associated with significant reduction of TA muscle twitch force, suggesting a reciprocal effect between TA and TS muscles. RM ANOVA showed a significant effect of tsDCS on sciatic nerve latency (*F* = 23.6, *P* < 0.001; *n* = 6). Latency of the short‐latency response was reduced during c‐tsDCS (7.8 ± 0.2 ms; *P* < 0.001), but increased during a‐tsDCS (14.9 ± 1.2 ms; *P* < 0.007) compared to baseline (11.8 ± 0.6 ms) (Fig. 3B, Holm–Sidak method). RM ANOVA also showed a significant effect of tsDCS on latency of the long‐latency response (*F* = 9.7, *P* < 0.002). Latency was reduced during c‐tsDCS (42.4 ± 4.2 ms; *P* < 0.03), but increased during a‐tsDCS (64.4 ± 3.6 ms; *P* < 0.04) compared to baseline (53.5 ± 2.6) (Fig. 3B; Holm–Sidak method). As spinal excitatory interneurons are arranged in separate microcircuit modules that can engage different motor units (Ampatzis et al. 2014), the current data suggest that tsDCS changes the configuration of spinal cord circuits. Thus, a faster and stronger muscle response could indicate that tsDCS altered spinal interneuron microcircuits to recruit faster motor units in response to the same test stimulation. Longer duration of c‐tsDCS (3 min) inhibited MLF‐evoked sciatic nerve potentials for 20 min after c‐tsDCS offset (an effect opposite that observed during stimulation, data not shown). However, following a‐tsDCS, potential amplitude was increased (data not shown). These data agree with our previous study (Ahmed 2011).

Next, in a different group of animals, a high‐frequency testing procedure was used (train of 5 pulses; frequency, 400 Hz; duration, 0.2 ms; intensity 0.2 mA). As shown in Figure 3C, this test stimulation produced one sciatic nerve response. Sciatic nerve responses and concurrent muscle twitch forces were increased during c‐tsDCS, but decreased during a‐tsDCS. In addition, RM ANOVA revealed significant effect of tsDCS on latency of sciatic nerve responses (*F* = 14.7; *P* < 0.001; *n* = 5) (Fig. 3D). Response latency was decreased during c‐tsDCS (12.6 ± 0.3 ms, *P* < 0.001), but was not changed during a‐tsDCS (14.4 ± 0.3 ms, *P* = 0.6) compared to baseline (14.6 ± 0.3) (Holm–Sidak method).

Longer tsDCS (3 min) interacted with the test stimulus to change the after‐effect outcomes. Sciatic nerve responses and muscle twitch forces were increased after c‐tsDCS and decreased after a‐tsDCS (data not shown). In general, this is consistent with previous findings using a high‐frequency test procedure (Ahmed and Wieraszko 2012).

### Experiment 2: Effects of tsDCS on LFT excitability

#### a‐tsDCS modified synaptic and nonsynaptic spinal and sciatic potentials, as well as muscle twitch force

It is critical to recognize changes in spinal cord white matter excitability versus synaptically mediated responses. Thus, the LFT was stimulated at a rostral location, field potentials were recorded from both LFT and the sciatic nerve, and muscle twitch force was recorded from TA and TS muscles (Figs. 1B and 2B). Spinal local traces were clearly separated into three waves. The first and second waves were designated tract CAPs because of their resistance to kynurenic acid injection (data not shown; see also Ahmed 2013a).

**Figure 4.**
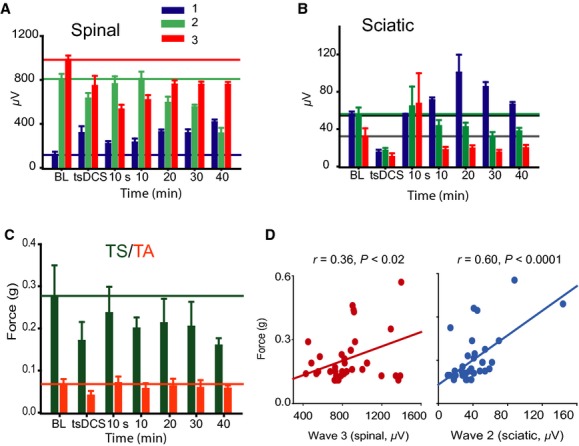
a‐transspinal direct current stimulation modified activation of synaptic and nonsynaptic responses. Sciatic nerve potentials and muscle responses were evoked by LFT stimulation. (A) Comparison of mean responses of spinal waves. (B) Comparison of mean responses of sciatic waves. (C) Comparison of mean twitch force of TS and TA muscles. All lines depict the average response at baseline (BL). (D) Of the three spinal waves (1, 2 and 3), only wave 3 significantly correlated with muscle twitch force. Similarly, only sciatic wave 2 significantly correlated with muscle force. Data represent mean ± SEM.

RM ANOVA revealed significant effects of a‐tsDCS on the first (*F* = 28.1, *P* < 0.001), second (*F* = 3.6, *P* < 0.003), and third spinal waves (*F* = 20.9, *P* < 0.001), as shown in Figure 4A (*n* = 8). Compared to baseline, the first wave was increased during and across the 40 min after a‐tsDCS. The second wave was reduced during and 20 to 40 min after a‐tsDCS. The third wave was reduced during and across the 40 min after a‐tsDCS.

There were significant effects of a‐tsDCS on the first (*F* = 7.4, *P* < 0.001), second (*F* = 3.1, *P* < 0.02), and third sciatic waves (*F* = 5.6. *P* < 0.001), as shown in Figure 4B. Compared to baseline, the first wave was reduced during a‐tsDCS, reverted to baseline value immediately after offset, then increased from 10 to 40 min after offset. The second wave was reduced during a‐tsDCS, reverted to baseline immediately afterward, then decreased from 10 to 40 min. The third wave was reduced during a‐tsDCS, increased immediately afterward, then decreased from 10 to 40 min.

There were significant effects of a‐tsDCS on TS (*F* = 6.6, *P* < 0.001) and TA muscle twitch force (*F* = 8.3, *P* < 0.001), as shown in Figure 4C. Compared to baseline, TS twitch force was reduced during a‐tsDCS, reverted to baseline values immediately after offset, then decreased from 10 to 40 min after offset. TA twitch force was reduced during a‐tsDCS, but showed no lasting effect. Pearson correlation was used to identify the spinal and sciatic waves that evoked muscle contraction. Muscle twitch force showed significant positive correlations with the third spinal and second sciatic waves (Fig. 4D), confirming that these waves are synaptically transmitted.

#### C‐tsDCS modified synaptic and nonsynaptic spinal and sciatic potentials and muscle twitch force

**Figure 5.**
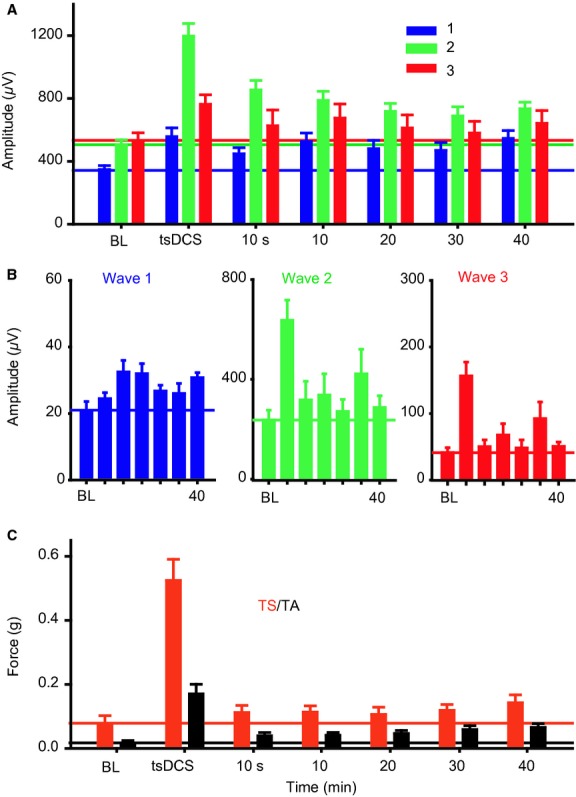
c‐transspinal direct current stimulation modified activation of synaptic and nonsynaptic responses. (A) Summary plot showing all spinal waves that were significantly amplified by c‐tsDCS. Horizontal lines mark the averages at baseline (BL). (B) Summary plots showing that sciatic nerve waves were amplified during and after c‐tsDCS. (C) Summary plot showing that TS and TA twitch force was significantly increased during and after c‐tsDCS. All values above the horizontal lines were statistically significant. Data represent mean ± SEM.

There were significant main effects of c‐tsDCS on the first (*F* = 6.2, *P* < 0.001), second, (*F* = 16.3, *P* < 0.001) and third spinal waves (*F* = 12.1, *P* < 0.001), as shown in Figure 5A (*n* = 7). All three spinal waves were increased during c‐tsDCS and after offset. Similarly, there were significant main effect of c‐tsDCS on the first (*F* = 7.1, *P* < 0.001), second (*F* = 9.2, *P* < 0.001), and third (*F* = 13.5, *P* < 0.001) sciatic waves, as shown in Figure 5B. All three sciatic waves were increased during and after c‐tsDCS. Finally, c‐tsDCS significantly increased responses of TS muscle (*F* = 25.8, *P* < 0.001) and TA muscle (*F* = 16.0, *P* < 0.001) across 40 min, as shown in Figure 5C. In summary, c‐tsDCS augmented both white matter and synaptic excitability in the spinal cord.

### Experiment 3: Local subthreshold DC stimulation had long‐lasting effects on sciatic nerve excitability

As described above, subDCS has immediate‐ and after‐effects on excitability of spinal cord white matter. However, a simpler model was needed to identify specific factors mediating subDCS effects on axonal excitability. We used a sciatic nerve preparation because it contains only axons with predictable directions. In addition, the sciatic nerve can be easily oriented relative to different DC electrode arrangements. Here, we tested three DC arrangements relative to the sciatic nerve: a single electrode, two parallel electrodes, and two perpendicular electrodes.

### Single DC electrode stimulation

#### Test stimulation at the nerve segment in front of the DC electrode

**Figure 6.**
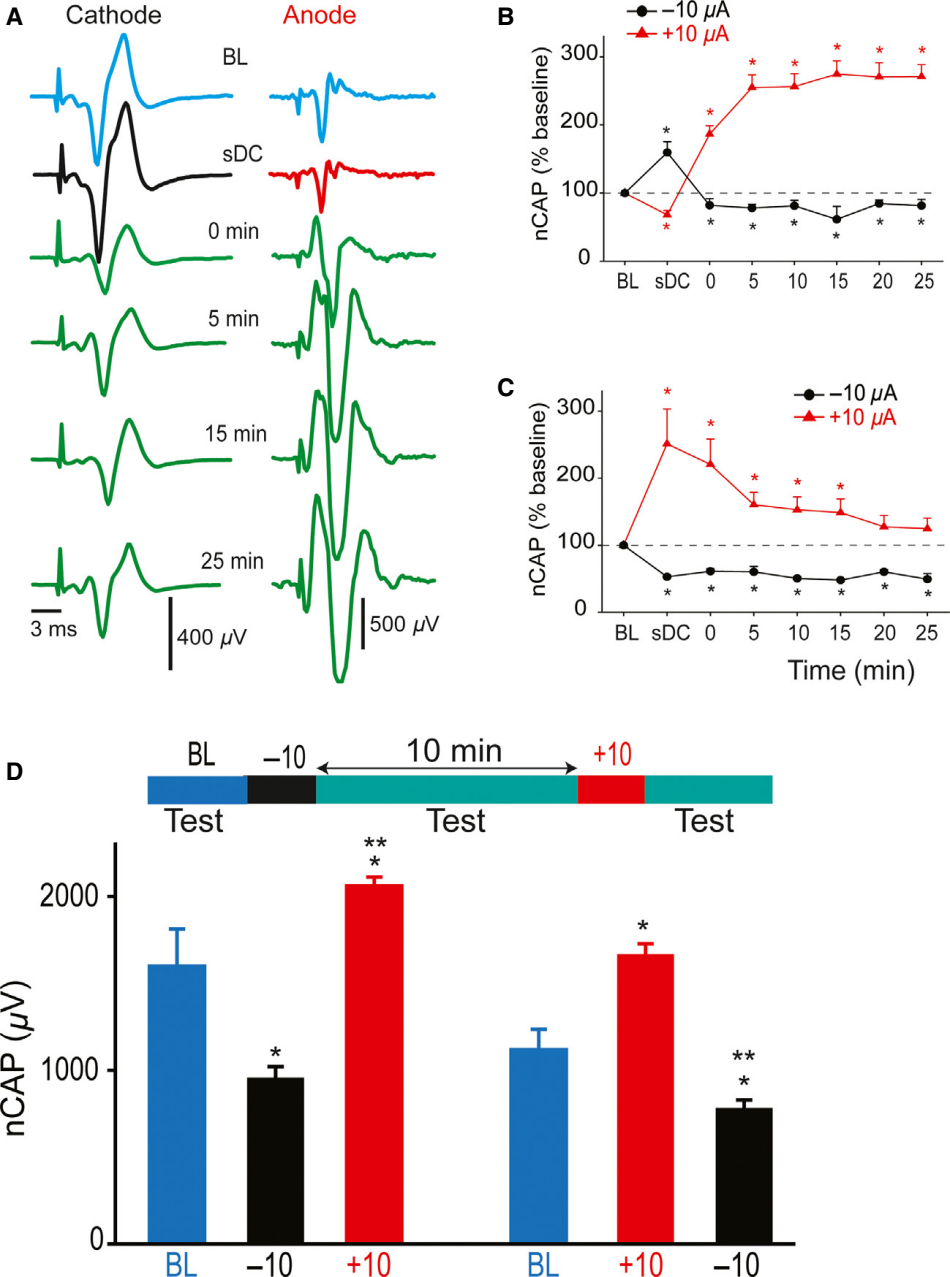
Effects of single‐sub‐DC electrode stimulation on sciatic nerve excitability. (A) Examples of CAP traces recorded from nerve segment lie in front of the sub‐DC electrode. (B) Test stimulation at the nerve segment in front of the subDCS electrode. a‐sDCS enhanced nerve excitability for 25 min, and c‐sDCS depressed nerve excitability for 25 min following current offset. Note that the direction of excitability was reversed after subDCS compared to during subDCS. (C) Test stimulation proximal to the DC electrode. a‐subDCS enhanced nerve excitability for 15 min, and c‐subDCS depressed nerve excitability for at least 25 min following current offset. (D) Nerve excitability changes could be reversed by applying the opposite polarity. Top: Experimental outline. Bottom: Summary plot showing that applying a current with opposite polarity could reverse the effect of the previous current. As evident in the summary plot, a‐subDCS not only reversed c‐subDCS‐induced depression, but its effect significantly exceeded that of baseline. Similarly, c‐subDCS not only reversed a‐subDCS‐induced enhancement, but reduced CAP significantly from baseline. Data represent means ± SEM. *P < 0.05 from baseline; **P < 0.05 from the corresponding subDCS condition.

We first tested the effect of single‐electrode subDCS on the excitability of the sciatic nerve segment in front of the electrode. RM ANOVA detected a significant main effect of anodal‐subDCS (a‐subDCS) on CAP (*F* = 27.6, *P* < 0.001, *n* = 6). The amplitude of the CAP was decreased during a‐subDCS (*P* < 0.001), but increased for at least 25 min after offset (Holm–Sidak method, *P* < 0.001), as shown in Figure 6A and B. Kruskal–Wallis one‐way RM ANOVA on Ranks revealed a significant main effect of cathodal subDCS (c‐subDCS) on CAP (H = 72.6, *P* < 0.001; *n* = 5). The amplitude of the CAP was increased during cathodal subDCS (Tukey test, *P* < 0.001), then decreased for at least 25 min after offset (Tukey test, *P* < 0.05) (Fig. 6A and B).

#### Proximal test stimulation

RM ANOVA showed significant main effects of a‐subDCS (*F* = 31.9, *P* < 0.001; *n* = 7) and c‐subDCS (*F* = 20.3, *P* < 0.001, *n* = 6), as shown in Figure 6C. During a‐subDCS, the amplitude of the CAP was increased, and this effect persisted for 15 min after offset (Holm–Sidak method, *P* < 0.01). During c‐subDCS, the amplitude of the CAP was decreased, and this effect persisted for at least 25 min after offset (Holm–Sidak method, *P* < 0.01).

Latency of sciatic nerve potentials was also measured in these experiments. There was a significant increase in latency during a‐subDCS (2.25 ± 0.07 ms) compared to baseline (1.6 ± 0.1 ms) (paired t‐test, *P* = 0.007) and a significant decrease in latency during c‐subDCS (1.7 ± 0.1) compared to baseline (2.1 ± 0.1) (paired t‐test; *P* = 0.02).

#### Reversibility of subDCS effects

Next, we tested whether the long‐lasting effects of subDCS could be reversed by applying the opposite subDCS polarity. Proximal test stimulation was used in these experiments. In the first group of experiments (*n* = 5), c‐subDCS was applied for 3 min, then 15 min after its offset, a‐subDCS was applied for 3 min, as shown in Figure 6D. RM ANOVA showed a significant effect (*F* = 25.3, *P* < 0.001) because c‐subDCS decreased CAP (Holm–Sidak method, *P* < 0.001), and subsequent application of a‐subDCS increased CAP relative to baseline (*P* < 0.003) and to c‐subDCS (*P* < 0.01) (Holm–Sidak method). In the second group of experiments (*n* = 5), a‐subDCS was applied first. RM ANOVA showed a significant effect (*F* = 33.9, *P* < 0.001) because a‐subDCS increased CAP (Holm–Sidak method, *P* < 0.001), and a subsequent application of c‐subDCS (782.4 ± 46.3 *μ*V) decreased CAP compared to baseline (*P* < 0.05) and a‐subDCS (*P* < 0.001) (Holm–Sidak method). Note that the opposite polarity of subDCS did not simply reverse the effect of the previous polarity, but it induced a change that was significantly different from baseline.

### Two sDC electrodes parallel to the sciatic nerve

#### Immediate effects

##### Proximal test stimulation: nerve centered between electrodes

**Figure 7.**
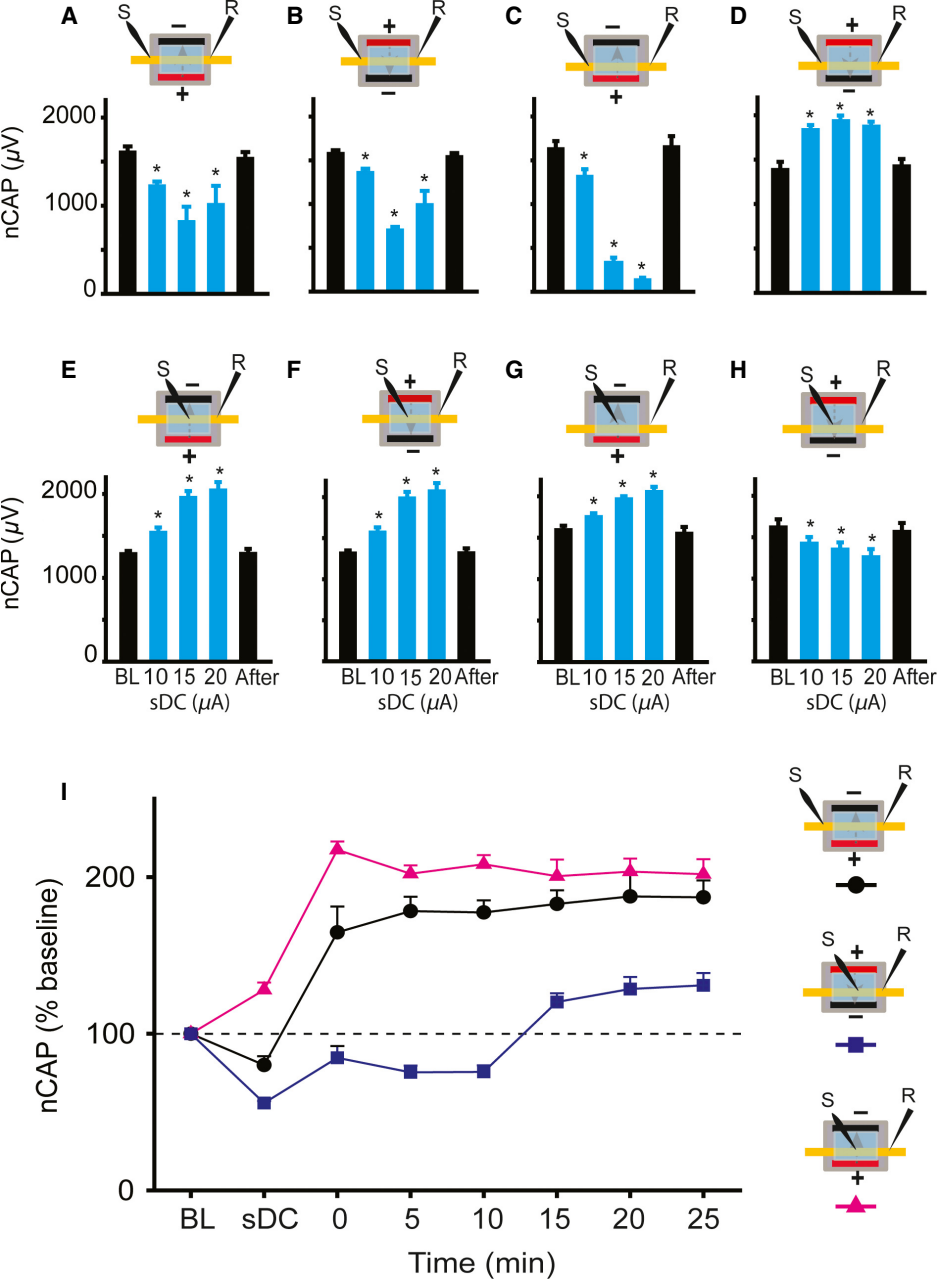
Effects of parallel subDC electrode arrangement on sciatic nerve excitability. In A–D, between‐electrode test stimulation was used. (A,B) When the nerve was centered between the two sDC electrodes, both lateral to medial subDCS and medial to lateral subDCS caused significant inhibition. (C) When the nerve was moved closer to the anode, the inhibition was enhanced. (D) When the nerve was moved closer to the cathode, the excitability was enhanced. In E–H, between‐electrode test stimulation was used. (E,F) When the nerve was centered between the two sDC electrodes, both lateral to medial subDCS and medial to lateral subDCS caused enhancement that was linearly related to current strength. (G) When the nerve was moved closer to the anode, subDCS significantly enhanced excitability. (H) Conversely, when the nerve was moved closer to the cathode, subDCS significantly inhibited nerve excitability. (I) Long‐lasting effects of parallel sDC electrode arrangement on sciatic nerve excitability. Insets on the right of the figure show the different experimental setup. When the nerve was centered between the two sDC electrodes, lateral to medial subDCS caused inhibition during subDCS. However, nerve excitability increased significantly after subDCS offset, and this enhancement lasted at least 25 min. When the nerve was moved closer to the cathode, nerve excitability decreased significantly during subDCS and for 10 min after subDCS offset, then increased significantly from 15 to 25 min. When the nerve was moved closer to the anode, nerve excitability increased significantly during sDC and lasted at least 25 min after subDCS offset. Data represent means ± S.E.M. *P < 0.05 relative to baseline.

In this group of experiments (*n* = 6), the nerve was centered between the two DC electrodes, and sub‐DC current was passed in the lateral to medial direction (Fig. 2C). RM ANOVA showed significant effects on CAP (*F* = 7.5, *P* < 0.001) (Fig. 7A). Compared to baseline, the amplitude of the CAP decreased as a function of subDCS strength (*P* < 0.01), then reverted to baseline values after subDCS offset (Holm–Sidak method, *P* > 0.05). Passing the current in the opposite direction (medial to lateral) had similar effects. RM ANOVA showed significant effects on CAP (*F* = 34.6, *P* < 0.001) (Fig. 7B). Compared to baseline, the amplitude of the CAP decreased (*P* < 0.01), then reverted to baseline values after subDCS offset (Holm–Sidak method, *P* > 0.05).

##### Proximal test stimulation: nerve closer to one electrode

The nerve was brought closer to either the anode or the cathode (1 mm relative to 4 mm), and a series of subDCS strengths were passed between the electrodes. RM ANOVA showed significant effects when the nerve was closer to a‐subDCS (*F* = 152.2, *P* < 0.001; *n* = 5) (Fig. 7C). Compared to baseline, the amplitude of the CAP decreased (Holm–Sidak method, *P* < 0.001), then reverted to baseline value after subDCS offset (*P* > 0.05). In the same experiments, the current direction was switched so that the nerve was closer to the cathode. RM ANOVA showed significant effects when the nerve was closer to c‐subDCS (*F* = 49.0, *P* < 0.001) (Fig. 7D). Compared to baseline, the amplitude of the CAP increased (Holm–Sidak method, *P* < 0.001), then reverted to baseline values after subDCS offset (Holm–Sidak method, *P* > 0.05).

##### Test stimulation at the nerve segment between DC electrodes

Figure 7E illustrates the effect produced by passing various strengths of subDCS in the lateral to medial direction while the nerve was positioned equidistant between the two DC electrodes. RM ANOVA showed significant effects of subDCS (*F* = 72.4, *P* < 0.001; *n* = 5). At all strengths, CAP was increased compared to baseline (Holm–Sidak method, *P* < 0.001), then reverted to baseline values following subDCS offset (Holm–Sidak method, *P* > 0.05). Passing the current in the opposite direction (medial to lateral) produced a similar effect on CAP (Fig. 7F). RM ANOVA showed a significant effect of subDCS (*F* = 72.4, *P* < 0.001). At all strengths, CAP increased compared to baseline (Holm–Sidak method, *P* < 0.001), then reverted to baseline values after subDCS offset (Holm–Sidak method, *P* > 0.05).

Figure 7G shows the effect produced by bringing the nerve closer to the a‐subDCS electrode (1 mm vs. 4 mm). RM ANOVA showed significant effects of a‐subDCS (*F* = 33.6, *P* < 0.001; *n* = 5). Compared to baseline, CAP increased (*P* < 0.001), then reverted to baseline values after a‐subDCS offset (Holm–Sidak method, *P* > 0.05). Figure 7H illustrates the effect produced by bringing the nerve closer to the c‐subDCS electrode. RM ANOVA showed significant effects of c‐subDCS (*F* = 10.4, *P* < 0.001). Compared to baseline, CAP decreased (*P* < 0.001), then reverted to baseline value after c‐subDCS offset (Holm–Sidak method, *P* > 0.05).

#### Persistent after‐effects of parallel subDCS arrangement

To examine the after‐effects of the parallel subDCS electrode arrangement, subDCS (15 *μ*A) was passed between the two polarizing electrodes for 3 min. The centered nerve preparation always produced a persistent increase in nerve excitability regardless of the location of test stimulation; therefore, results from only one of these experiments will be shown here (lateral to medial subDCS; nerve centered). RM ANOVA showed significant effects of subDCS on excitability of the centered nerve preparation (*F* = 47.3, *P* < 0.001; *n* = 6). As expected, CAP decreased during subDCS (*P* < 0. 05), but increased at all time points following subDCS offset (Holm–Sidak method, *P* < 0.001) (Fig. 7I). Next, the after‐effects of subDCS were tested when the nerve was brought closer to the cathode (Fig. 7I). RM ANOVA showed significant effects of subDCS (*F* = 65.9, *P* < 0.001; *n* = 5). Compared to baseline, CAP was decreased during subDCS (*P* < 0.001) and for 0–10 min after subDCS offset (Holm–Sidak method, *P* < 0.001), then increased from 15 to 25 min (Holm–Sidak method, *P* < 0.001). Finally, when the nerve was placed closer to anode, RM ANOVA showed a significant effect of subDCS on CAP (*F* = 68.3, *P* < 0.001; *n* = 5). Compared to baseline, CAP increased during subDCS and at all time points after sDC offset (Holm–Sidak method, *P* < 0.001) (Fig. 7I).

#### Two subDCS electrodes perpendicular to the sciatic nerve

##### Proximal test stimulation

**Figure 8.**
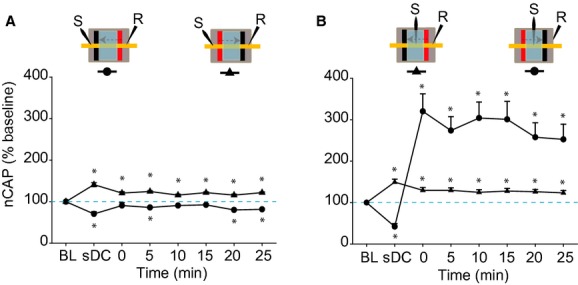
Long‐lasting effects of perpendicular subDCS electrode arrangement on sciatic nerve excitability. (A) Proximal test stimulation. Distal to proximal subDCS caused inhibition that was evident 25 min after current offset. Proximal to distal subDCS caused enhancement that lasted at least 25 min after current offset. (B) Between‐electrode test stimulation. Distal to proximal subDCS caused enhancement that lasted at least 25 min. Proximal to distal subDCS caused inhibition during subDCS, but significantly enhanced CAP after subDCS offset. Insets on the top of the figure show the experimental setup. Data represent means ± SEM. *P < 0.05 relative to baseline.

To test the long‐term effects of subDCS in the distal to proximal direction, a current of 5 *μ*A was applied for 3 min. Proximal test stimulation was used in these experiments (Fig. 8A). To facilitate comparison, data were expressed as a percentage of baseline. RM ANOVA showed a significant effect (*F* = 13.6, *P* < 0.001; *n* = 5). Compared to baseline, CAP was decreased (61.9 ± 1.8%). Following subDCS offset, CAP was decreased at three time points (5, 20, and 25 min; *P* < 0.001) and showed nonsignificant but numerical decreases at the other three time points (0, 10, and 15 min; *P* > 0.05) (Holm–Sidak method) (Fig. 8A). Next, the opposite subDCS direction (proximal to distal; *n* = 5) was tested. RM ANOVA showed a significant effect (*F* = 18.6, *P* < 0.001). Compared to baseline, CAP was increased during subDCS and at all time points afterward (*P* < 0.001, Holm–Sidak method) (Fig. 8A).

##### Between‐electrode test stimulation

Between‐electrode test stimulation was used to test the long‐term effects of different subDCS directions on nerve excitability. RM ANOVA showed significant effects of proximal to distal subDCS on CAP (*F* = 37.1, *P* < 0.001; *n* = 6; Fig. 8B). Compared to baseline, CAP amplitude decreased during subDCS (*P* < 0.01), then rebounded and increased at all time points after offset (*P* < 0.001; Holm–Sidak method). RM ANOVA showed significant effects of distal to proximal subDCS (*F* = 13.9, *P* < 0.001; *n* = 5). Compared to baseline, CAP amplitude increased during subDCS (*P* < 0.001) and remained increased at all time points following offset (*P* < 0.001, Holm–Sidak method). Overall, these findings emphasize that the effects of subDCS are dependent on the polarity of the subDCS electrode closest to the test point (Fig. 8A), and more importantly, on the current direction (Fig. 8B).

## Discussion

In this study, the effects of tsDCS on synaptically mediated evoked responses were dependent on the pattern of test stimulation. Specifically, amplitude of synaptically mediated evoked responses increased during c‐tsDCS and decreased after offset. We believe that this modulatory effect of c‐tsDCS was revealed by low‐frequency test stimulation. However, high‐frequency test stimulation prolonged the enhancement of synaptically‐evoked responses by c‐tsDCS. These results are in agreement with previous evidence suggesting interactions between activity and DCS (Nitsche et al. 2007; Ahmed and Wieraszko 2012; Ahmed 2013b). Understanding this interaction will be valuable for designing and interpreting behavioral experiments using tsDCS or transcranial DCS.

As shown in Figure 3, baseline MLF stimulation evoked TA muscle twitch and concurrently reduced the background tension of TS muscle, which showed a superimposed TS twitch. However, during c‐tsDCS, the same test stimulation evoked contraction of both TA and TS muscles. This shows that c‐tsDCS can change the spinal circuitry activation pattern. Moreover, c‐tsDCS shortened the first response latency by 4 ms, and a‐tsDCS prolonged latency by 3.1 ms (Fig. 1C). Assuming that conduction time would change similarly as in sciatic nerve (by about 0.5 ms), the remainder of the change in the latency could be attributed to changes in synaptic pathways. This suggests that tsDCS configures these responses via a pathway of at least three synapses. The effect of tsDCS on the latency of the delayed response following a single‐teststimulus (Fig. 3A and B) was altered considerably. Specifically, c‐tsDCS shortened the delay time by 10.7 ms, and a‐tsDCS increased it by 10.9 ms. The length of that increase indicates an involvement of at least 10 synapses. Thus, these data suggest that spinal circuitry is readily dynamic, and local spinal excitability is very important in shaping the response to supraspinal inputs. This raises the question of how local spinal excitability is naturally attuned to accurately assemble supraspinal commands.

Spinal cord circuits can be configured to produce reflexive, rhythmic (e.g., walking), or voluntary movements. Supraspinal systems and sensory inputs play an important role in modulating spinal circuits to adapt to a particular type of movement. For example, increasing the intensity of inputs from the mesencephalic locomotor region to the central pattern generator patterning circuit shifts the gait pattern from slow walking (alternating pattern) to galloping (synchronous pattern). This shifts the activity of hindlimb muscles from out‐of‐phase (alternating) to in‐phase (co‐contraction). The pattern of locomotor activity largely depends on the excitability of spinal inhibitory interneurons. Therefore, to explain the present findings, we propose that c‐tsDCS inhibits spinal inhibitory interneurons. This allows production of synchronous activity of antagonistic motor neurons, as shown in this study (Fig. 3). Thus, we propose that online c‐tsDCS could be used to suppress spinal cord inhibitory circuits to allow a desirable motor task (e.g., jumping).

Low‐frequency test stimulation showed that longer duration a‐tsDCS caused amplification of MLF‐evoked sciatic nerve responses after current offset, however, LFT‐evoked sciatic nerve responses were decreased after the same protocol. This difference indicates that a‐tsDCS affects local tract excitability. This is supported by the observation that the second wave (Fig. 4A) was decreased after a‐tsDCS. Depressed excitability of the LFT would affect its response to test stimulation. Low‐frequency test stimulation also revealed that potentials with faster conduction velocity (i.e., the first wave) were amplified by a‐tsDCS. This wave is most likely a result of direct stimulation of afferent fibers, as it had high conduction velocity and was not associated with muscle twitches. This suggests that size of neurons may be a factor determining the direction of the plastic change induced by a‐tsDCS. c‐tsDCS produced opposite effects on spinal tracts and afferent fiber potentials. Overall, these findings underscore the intricate responses of spinal cord to tsDCS. In addition, these results show the importance of test procedure parameters in interpreting the overall effects of subDCS.

This study demonstrated that subDCS can induce short‐ and long‐lasting changes in the excitability of nerve fibers. During stimulation, single electrode anodal subDCS increased nerve excitability in segments further away from the electrode and decreased excitability of segments overlaying the electrode, and cathodal subDCS had opposite effects. It should be emphasized that in single‐electrode application of subDCS, the return electrode was attached to abdominal skin. However, in exploratory experiments (data not shown), the return electrode was attached to the paw, tail, or abdominal skin on the same or opposite side of the body. This did not alter the effects of the sciatic DC electrode. This indicates that the return electrode position is only meaningful if it is placed near or in direct contact with neural tissue, as shown in the parallel and perpendicular electrode experiments. Regardless of the site of test stimulation, after current offset, anodal subDCS caused long‐lasting increases in nerve excitability, and cathodal subDCS caused long‐lasting decreases in nerve excitability. The results shown in Figure 6B indicate that long‐lasting effects were induced for the most part by an independent mechanism of immediate excitability changes. Based on these findings, application of subDCS seems to immediately invoke nonhomeostatic intrinsic plasticity, followed by a form of homeostatic intrinsic plasticity. These forms of intrinsic neuronal plasticity are extremely important in health and disease. Nonhomeostatic intrinsic plasticity is co‐induced with synaptic plasticity by LTP‐inducing stimulation and is thought to complement synaptic changes (see review: Zhang and Linden 2003). Thus, subDCS‐induced changes in axonal excitability can modulate the likelihood of synaptic plasticity. SubDCS could elicit these modulations by changing action potential threshold (as shown in the present study) or firing mode of the postsynaptic neuron. As homeostatic responses always follow a long period of altered neuronal activity, the after‐effects of subDCS seem to be a homeostatic response of intrinsic excitability. The difference in the sciatic nerve experiments, however, was that the imposed change in excitability was subthreshold and was not accompanied by actual neuronal activity. This indicates that altered membrane potential is the key factor in modulating intrinsic homeostatic plasticity. Given that homeostatic plasticity is abnormal in many CNS disorders (Beck and Yaari 2008), subDCS could be a valuable tool to normalize altered homeostatic excitability in disorders such as traumatic brain injury (Howard et al. 2007), epilepsy (Sanabria et al. 2001; Wellmer et al. 2002), pain (Tan et al. 2006; Wang et al. 2007), and addiction (Moussawi et al. 2011).

One of the most important findings in this study was that the long‐lasting effects of subDCS were reversible. Not only did subDCS of one polarity reverse the effects of the opposite polarity, but it significantly changed CAP amplitude relative to baseline. This has three implications: 1) subDCS did not damage the nerve fibers, 2) the mechanism(s) underlying the effects of subDCS can be changed in either direction (increase or decrease), similar to synaptic mechanisms of plasticity (Weragoda et al. 2004), and 3) increases and decreases are mediated by the same mechanisms. These findings also mimic the bidirectional activity‐dependent changes observed due to the intrinsic properties of neurons (Turrigiano et al. 1994).

It is known that an anodal electrode would hyperpolarize the nerve segment in front of the electrode and depolarize distant segments, acting as a virtual cathode distally (Roth 1994), and the opposite effects would be evoked by a cathodal electrode (Merrill et al. 2005). This principle can explain the immediate effects of the perpendicular arrangement of subDCS electrodes. During proximal test stimulation, when the cathodal electrode was closer to the test electrode, CAP was reduced, presumably due to hyperpolarization of the distant nerve segment. Conversely, when the anodal electrode was closer to the test stimulation site, CAP was increased. During the between‐electrode test stimulation procedure, CAP was increased when an anodal electrode intervened between the testing and recording electrodes and decreased when a cathodal electrode intervened.

Apparently, electrodes touching the nerves affect excitability differently from electrodes making indirect contact with nerves through a conductive fluid. This was evident in results obtained from parallel electrodes (Fig. 7). c‐subDCS that was closer to the nerve increased excitability at the distant segment and reduced it at the electrode. The reverse occurred with a‐subDCS. It is possible that ions in the fluid accumulated around the electrodes, which had opposite charges, reversing their effects on the axons. Therefore, tissue surrounding the anode would adopt a cathodal pattern of excitability and vice versa (Fig. 7). It should be also noted that even nerves aligned exactly equidistant between the two parallel electrodes showed a response to polarization. Rearrangement of charges by polarization can cause equipotential at the center between the parallel electrodes. However, the effect on axonal physiology would be greatly affected by the type of charges that were rearranged by polarization. Many charged proteins and ions can move in response to polarization and cause changes that may or may not depend on their charges. For example, the screening effect of extracellular Ca^2+^ can be disturbed by polarization. This very important factor can change excitability of axons (Del Castillo and Katz 1954).

The after‐effects of single‐electrode subDCS are similar in the brain (Nitsche and Paulus 2000; Antal et al. 2004), spinal cord (Ahmed 2011; Ahmed and Wieraszko 2012), and peripheral nerves (present study). This suggests common underlying mechanisms, which seem to be dependent upon the polarity of the field but not the topography of neurons relative to the field. Applied electrical fields are known to cause charged receptor asymmetries (e.g., acetylcholine and epidermal growth factor receptors) (Jaffe 1977; Poo and Robinson 1977; Poo et al. 1978). This is most likely due to an electro‐osmosis phenomenon (McLaughlin and Poo 1981), which induces fluid flow near the cell membrane. Electro‐osmosis draws negatively charged molecules to the cathode electrode. If this happens intracellularly, negatively charged proteins, electrolytes, and amino acids, which maintain the resting membrane potential, could be redistributed to accumulate in hyperpolarized regions of the axon, leaving other regions depolarized. Electro‐osmosis or electrophoresis can rearrange charged molecules in the membrane, cytoplasm, or extracellular space, which in turn can cause long‐lasting changes in axonal excitability. Interestingly, the duration needed to induce long‐lasting changes in axonal excitability in this study (>1.5 min) is similar to that needed to induce substantial asymmetry in receptor distribution (1–2 min) (Jaffe 1977; Jaffe and Nuccitelli 1977; Poo 1981).As the sodium pump is a charged molecule (Morth et al. 2007), its redistribution by subDCS could help to mediate the long‐lasting effects of subDCS.

In conclusion, the current comprehensive study showed the neurophysiological effects of DCS on nervous tissues. Effects of DCS on synaptically mediated responses were determined by current polarity, neural activity and activation rate, duration of stimulation, and temporal profile (during vs. after stimulation). Effects on axonal excitability were determined by polarity, duration of stimulation, temporal profile (during vs. after stimulation), orientation of axon relative to current direction, current direction relative to action potential propagation direction (Fig. 6B), and distance from the DC electrode. Another influential factor was the local environment surrounding the nervous tissue. In addition, this study and previous work from our lab (Ahmed 2011) revealed that the strength of the DCS is important for both synaptically and nonsynaptically mediated responses. Finally, the location of the reference electrode on the neural tissue was also an important factor. Therefore, this study identified numerous factors that should be considered in interpreting results of DCS. These factors are critical in designing interventions using DCS and can be used to predict behavioral effects of DCS.

In addition, an important finding in this study was that the neuronal structures located directly under the electrode respond differently during DCS than the structures surrounding the electrode (Fig. 5). Although this was not directly tested, these data suggest that muscles with motor neuron pools located caudal to the tsDCS electrode should have a different response from those with motor neuron pools under the electrode. This issue could be significant for clinical translation due to the relatively larger size of humans. Finally, one major conclusion in our study is that DCS mainly affects intrinsic excitability to drive induction of synaptic plasticity depending on activity‐based rules. This could be a significant determining factor in using DCS to modulate different types of learning. For example, intrinsic forms of plasticity are believed to be more involved in nondeclarative learning (e.g., motor learning) (Zhang and Linden 2003). In this case, electrode size, location, and polarity of applied current could be major factors in determining the modulation of learning. Declarative learning, in which synaptic plasticity plays a greater role, could be more influenced by activity (i.e., performing the task) during DCS.

## Conflict of Interest

None declared.
